# Efficiency *vs.* stability: dopant-free hole transporting materials towards stabilized perovskite solar cells

**DOI:** 10.1039/c9sc01184f

**Published:** 2019-05-20

**Authors:** Kasparas Rakstys, Cansu Igci, Mohammad Khaja Nazeeruddin

**Affiliations:** a Group for Molecular Engineering of Functional Materials , Institute of Chemical Sciences and Engineering , École Polytechnique Fédérale de Lausanne , CH-1951 Sion , Switzerland . Email: k.rakstys@uq.edu.au ; Email: mdkhaja.nazeeruddin@epfl.ch

## Abstract

In the last decade, perovskite solar cells have been considered a promising and burgeoning technology for solar energy conversion with a power conversion efficiency currently exceeding 24%. However, although perovskite solar cells have achieved high power conversion efficiency, there are still several challenges limiting their industrial realization. The actual bottleneck for real uptake in the market still remains the cost-ineffective components and instability, to which doping-induced degradation of charge selective layers may contribute significantly. This article overviews the highest performance molecular and polymeric doped and dopant-free HTMs, showing how small changes in the molecular structure such as different atoms and different functional groups and changes in substitution positions or the length of the π-conjugated systems can affect photovoltaic performance and long-term stability of perovskite solar cells.

## Abstract 2

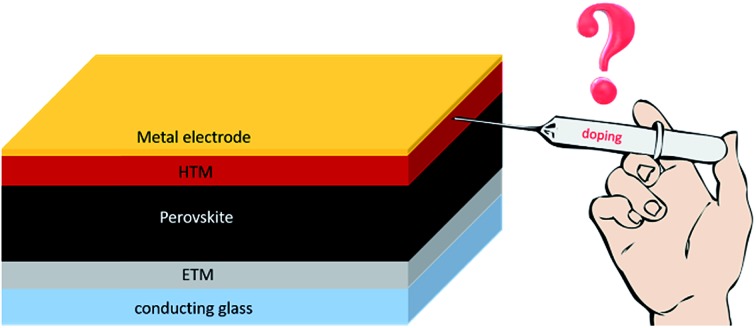
Doping of hole transporting materials typically increases the efficiency of perovskite solar cells but remains questionable for overall device stability.

## Introduction

Research on renewable energy is moving to the forefront and becoming indisputably relevant to the future security of energy needs. Among various renewable energy technologies, solar energy is an incredibly abundant and extremely clean source which has remained underexploited to date. Efficient and inexpensive energy conversion using solar cells is vital for global transition to a low-carbon society. Instead of the costly production of crystalline silicon, perovskite materials, which are inexpensive and abundant, are obtained by a relatively very simple synthesis process and are coated by conventional solution processing techniques.

The era of perovskite solar cells began only in 2009 with 3.8% PCE achieved by Miyasaka and his colleagues.^1^ Perovskite-based solar cells have rapidly became the hottest topic in photovoltaics due to their unique optical and electrical properties. Pioneering studies on the design of conventional perovskite solar cells have been reported in 2012 by Kim *et al.*^2^ and Lee *et al.*^3^ The efficiency of the perovskite solar cells has increased to 9.7% due to the dramatic improvement by utilizing spiro-MeOTAD as the hole transport material and mesoporous TiO_2_ as the electron transport material resulting in a solid-state meso-superstructured perovskite solar cell. This golden step has triggered the start of intensive studies of perovskite solar cells. The fast development of device engineering and perovskite composition has allowed improvement of current solar cells with over 24% power conversion efficiency certified by the NREL. In addition to improvement in the device performance, surpassing that of commercialized crystal semiconductors and other thin-film photovoltaic systems, the cost-effectiveness of perovskite materials and solution processed device manufacturing makes perovskite solar cell technology inevitable.

The organic–inorganic hybrid perovskite structure has crystals with general stoichiometry ABX_3_ and ideally has a cubic symmetry configuration wherein the A site is a monovalent organic methylammonium (CH_3_NH_3_^+^, MA^+^), formamidinium (NH_2_=CH_3_NH_2_^+^, FA^+^) or inorganic cation (Cs^+^ or Rb^+^), B is a divalent metal cation (Pb^2+^, Sn^2+^, or Ge^2+^) and *X* stands for a halide anion (I^–^, Br^–^, or Cl^–^).^4^,^5^ Although pioneering work in perovskite structures was conducted on methylammonium lead triiodide (CH_3_NH_3_PbI_3_) due to its excellent optoelectronic properties such as its broad absorption spectrum, high absorption coefficient,^1^ high charge carrier mobility,^6^ low exciton binding energy,^7^ and long electron and hole diffusion length,^8^ current research demonstrates that an increase in efficiency and stability of the perovskite-based solar cells is possible by combining more complex multi-cation and mixed-anion compositions of the perovskite absorber layer.

Although perovskite solar cells have achieved high power conversion efficiency, there are still several challenges limiting their industrial realization. Long-term stability is one of the important issues to be resolved. The perovskite layer is prone to rapid decomposition when the device is exposed to high temperature,^9^ humidity,^10^ oxygen,^11^ and full solar illumination.^12^ The degradation mechanism of methylammonium lead iodide in the presence of water reveals that the perovskite crystals easily decompose and are converted into the precursor PbI_2_ with irreversible degradation; this effect is even more pronounced in the presence of heat, an electrical field or UV light.^13^ On the other hand, water vapour exposure leads to a hydrated crystal phase (CH_3_NH_3_PbI_3_·H_2_O) due to the hydrophilic properties of perovskite, which is a reversible process only with dehumidification.^14^ Besides encapsulation of the cells, research efforts to improve long-term stability have been ongoing and are highlighted by interface modification with UV filtering caesium bromide (CsBr),^15^ modification of the composition of perovskite materials with the incorporation of caesium (Cs^+^) or rubidium (Rb^+^) cations in triple-cation perovskite solar cells,^16^,^17^ and engineering of interfaces with the creation of a 2D/3D perovskite combination which improves the stability and performance of the solar cell over more than one year without any loss in efficiency.^18^

The other bottleneck for real uptake in the market is the instability of the hole transporting material (HTM), to which doping-induced degradation of charge selective layers may contribute significantly, while other important key factors are the hygroscopic nature of HTMs, pinholes in the film morphology, humidity and thermally induced instability, interfacial degradation at the HTM/Au interface and undesirable chemical interactions of the HTM with the perovskite layer.^19^,^20^ In particular, wide bandgap HTMs such as spiro-OMeTAD typically require doping in order to achieve the necessary electrical conductivity, and the use of additives is problematic, since the hygroscopic nature of dopants makes the hole transporting layer highly hydrophilic leading to chemical degradation and negatively influences the stability of the entire device.^21^ One promising method to stabilize perovskite solar cells could be the appropriate choice of dopant-free and highly hydrophobic hole transporting materials, acting as a protective layer against moisture and preventing diffusion into the perovskite layer. Beside this, the hygroscopic nature of HTMs can be partially limited by device encapsulation.^10^ One of the possible solutions for thermal instability is using thermally and chemically stable metal–organic complexes. Due to their excellent hole transport properties, metal–organic complex HTMs have been incorporated into perovskite solar cells, but high PCE has not been achieved.

Another approach to enhance the stability is through interface engineering of HTMs. Introducing a thin (10 nm) chromium (Cr) layer between the spiro-MeOTAD and the top gold electrode reduced the dramatic device degradation associated with increased temperature. Basically, the addition of the Cr layer prevents gold migration through the HTM to the perovskite layer.^22^ Besides, it has been revealed that an integrated Al_2_O_3_ mesoporous buffer layer in the HTM prevents the metal electrode migration that occurs during the aging process and eliminates pin-hole induced instability of HTMs by protecting the perovskite layer.^23^ Despite promising research results obtained in a short period of time, the researchers of perovskite solar cells still have a keen interest in enhancing the long-term stability of HTMs to achieve a lasting solution.

Another key factor that plays a major role in the commercialization potential is the cost-ineffectiveness of HTMs. To date, spiro-OMeTAD and PTAA are the hole transporting materials of choice routinely employed in order to maintain the highest efficiency; however, their prohibitively high price hinders progress towards low-cost perovskite solar cell manufacturing and may contribute to more than 30% of the overall module price. The reason lies in the complicated multi-step synthetic procedure, which is affordable for small quantities but is hardly scalable.

### Architectures and operating mechanism

Hybrid lead halide perovskites can cumulate the function of light absorption with n- and p-type conduction.^24^,^25^ This ambipolar charge transport property of perovskite makes the design of PSCs quite versatile. The common device architectures are composed of five layers, which allows several combinations of materials: a transparent electrode, an n-type semiconducting electron transporting material (ETM), photoactive perovskite, a p-type semiconducting hole transporting material (HTM) and a metallic electrode. Depending on the structure of the n-type layer, PSCs can be classified into mesoscopic or planar heterojunction structures,^26^,^27^ which can both be further divided into two categories based on the position of the p-type and n-type selective contact materials. The solar cells could thus be considered n–i–p (conventional) and p–i–n (inverted) structures,^28^–^30^ where the perovskite is an intrinsic semiconductor, and light enters through the n-type or the p-type layer, resulting in four different device architectures. Although inverted perovskite devices typically have higher stability with hysteresis-free behavior, conventional devices have been demonstrated to be superior in terms of power conversion efficiency. Moreover, since the perovskites have been proved to be ambipolar semiconductors, device architectures of PSCs that employ only one of the two charge selective contacts, such as ETM-free or HTM-free PSCs, have been demonstrated.^18^,^31^–^33^ However, these typically fail to produce high PCE, and therefore it is generally agreed that both the electron and hole selective contact materials are vital to achieve high performance in PSCs.

**Fig. 1:**
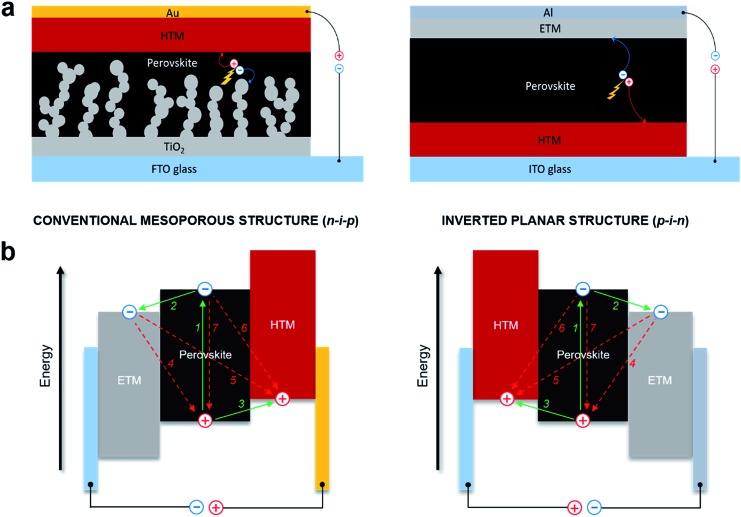
(a) Structure of conventional mesoscopic n–i–p and inverted planar p–i–n device configurations; (b) schematic illustration of energy levels and charge-transfer processes in different PSC architectures.

To date, the mesoscopic n–i–p and inverted p–i–n PSC device configurations have shown the highest performance reaching over 22%. In conventional mesoscopic architectures, the glass transparent electrode fluorine-doped tin oxide (FTO) is typically coated with a compact electron transporting layer (ETL) made from TiO_2_ and the additional mesoscopic TiO_2_ scaffold is used on top, following by infiltration with a photoactive perovskite material. A hole transporting layer (HTL) is deposited on top of the perovskite, followed by evaporation of a metallic top electrode, usually made of a high work function metal, such as gold, whereas the inverted planar architecture has swapped charge selective contacts as shown in Fig. 1a.^34^–^38^

The simplified operating mechanism and key charge-transfer processes of the typical PSCs are shown in Fig. 1b. Incident photons excite the perovskite light harvesting layer through the transparent electrode, leading to the photogeneration of electron–hole pairs in the material. Electrons are separated (1) from the holes and injected (2) into the conduction band (CB) of the electron transporting material before migrating to the anode. Meanwhile, the holes generated in the perovskite are transferred (3) to the HOMO level of the hole transporting material, before injection of the hole into the cathode. Electron and hole injections occur quite efficiently due to the high diffusion lengths of charge carriers. Undesirable charge transfer (CT) processes such as back CT recombination of the charge carriers at three ETM/perovskite/HTM interfaces also occur (4–6), including non-radiative recombination (7). In the meantime, it is expected that the kinetic processes of intrinsic radiative recombination,^7^ contact characteristics of the electrode,^39^ charge accumulation and diffusion lengths of the charge carriers^8^ greatly control the limit of photovoltaic conversion efficiency of the perovskite devices.

### Hole transporting layer

**Fig. 2:**
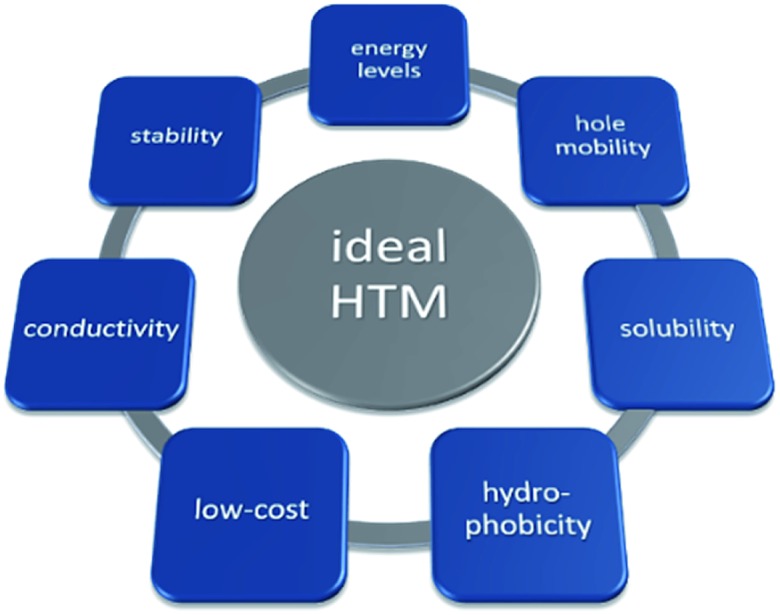
Schematic representation of summarized HTM properties required for PSCs in an ideal case.

HTMs are important active materials in PSCs, responsible for efficient hole extraction at the perovskite/HTM interface and prevention of undesirable charge recombination processes, leading to improvement in the performance of the device. For efficient device operation, well-aligned energy levels of the HTM with those of perovskite are crucial: the HOMO level must be less negative than that of perovskite for efficient hole injection, while the high-lying LUMO level will ensure low electron affinity enabling beneficial electron blocking properties. An ideal HTM candidate should also have high hole mobility to reduce losses during hole transport to the contact, as well as sufficient conductivity. The HTM has to be well soluble and should not require strong polar and protic solvents, as such solvents may dissolve the perovskite layer. Furthermore, since perovskites are known to be sensitive to moisture and oxygen, the hole transporting layer should act as protection against air and prevent the diffusion of external moieties; therefore high thermal and photochemical stability, as well as high hydrophobicity, are desirable properties of HTMs to resist degradation and ensure long-term durable PV operation. On the other hand, to ensure low-cost perovskite solar cell manufacturing, an ideal HTM candidate has to be easily affordable by simple synthetic schemes with a minimized number of steps and easy workup and purification procedures for cost-effective upscaling (Fig. 2).

A huge research interest directed at new HTM candidates has been devoted to gaining a better understanding of the relationship between the HTM and PSC performance. A large number and different types of HTMs have been reported so far, based on their organic^40^–^54^ and inorganic^55^–^58^ nature, of which organic HTMs can be further divided into small-molecules and conducting polymers.

To date, spiro-OMeTAD and PTAA are the hole transporting materials of choice routinely employed in order to maintain the highest efficiency; however, their prohibitively high price hinders progress towards low-cost perovskite solar cell manufacturing and may contribute to more than 30% of the overall module price. The reason lies in the complicated multi-step synthetic procedure, which is affordable for small quantities but is hardly scalable. Another key factor that plays a major role in the commercialization potential is the stability of the device. Additionally, wide bandgap hole transporting materials typically require doping in order to achieve the necessary electrical conductivity and the use of additives is problematic, since the hygroscopic nature of dopants makes the hole transporting layer highly hydrophilic leading to chemical degradation and negatively influences the stability of the entire device. One promising method to stabilize perovskite solar cells could be the appropriate choice of dopant-free and highly hydrophobic hole transporting materials, acting as a protective layer against moisture and preventing diffusion of external moieties.

**Fig. 3:**
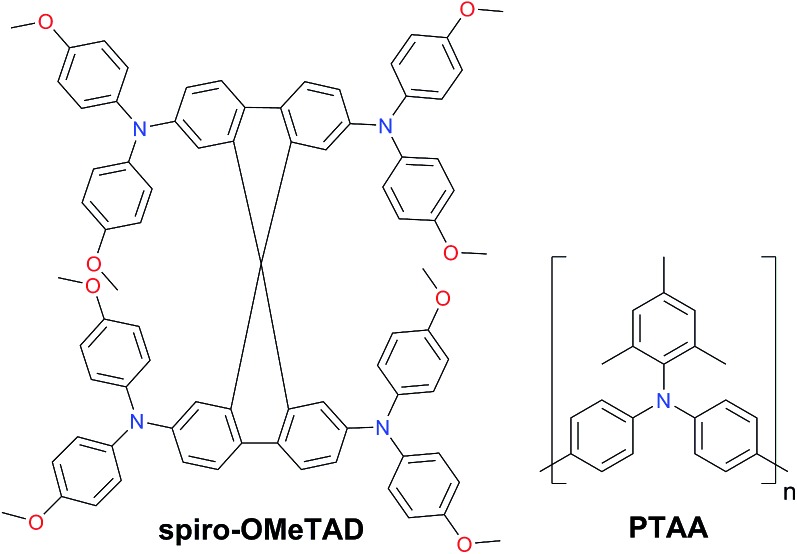
Chemical structures of 2,2′,7,7′-tetrakis-(*N*,*N*′-di-*p*-methoxyphenylamine)-9,9′-spirobifluorene (spiro-OMeTAD) and poly[bis(4-phenyl)(2,4,6-trimethylphenyl)amine] (PTAA).

Since the first report as a HTM in ssDSSCs two decades ago,^59^ the organic p-type semiconductor 2,2′,7,7′-tetrakis-(*N*,*N*′-di-*p*-methoxyphenylamine)-9,9′-spirobifluorene (spiro-OMeTAD) is still dominating the field and has been selected as the benchmark HTM to achieve high PCE values for PSCs. Spiro-OMeTAD has a HOMO level around –5.1 eV, exhibits a hole mobility in the range of 10^–5^ to 10^–4^ cm^2^ V^–1^ s^–1^ and has high solubility in organic solvents such as toluene or chlorobenzene, giving almost colorless thin films. However, the tedious multi-step synthesis of spiro-OMeTAD makes it prohibitively expensive and cost-ineffective. Typically, high-purity sublimation-grade spiro-OMeTAD is required to obtain high-performance devices. Moreover, it has been demonstrated to be a limiting factor for the long-term stability of the device (Fig. 3).^60^

Among the polymers, triphenylamine-based poly[bis(4-phenyl)(2,4,6-trimethylphenyl)amine] (PTAA) is the most efficient and holds the world record so far.^61^,^62^ The superior performance of PTAA arises from the exceptional hole mobility (10^–3^ to 10^–2^ cm^2^ V^–1^ s^–1^). However, it is extremely expensive reaching a price of ∼2000 $ per g.

Among the inorganic hole conductors, only very few examples achieving high efficiency have been shown in the literature. Due to the limited choice of suitable materials, inorganic HTMs remain quite an unexplored alternative to the organic ones. The three most interesting examples of this class are copper thiocyanate (CuSCN), copper gallium oxide (CuGaO_2_), and copper chromium oxide (CuCrO_2_). These wide bandgap semiconductors have high conductivity, suitable energy levels and good transparency. CuSCN has been studied by several research groups allowing improvement of the device performance from 12.4% to the very recent achievement over 20%.^63^–^66^ CuSCN-based devices also demonstrate improved stability under dry air and thermal annealing. Solution-processed delafossite phase CuGaO_2_ nanoplates were employed as the hole transporting layer yielding devices with an efficiency of 18.5% and significantly improved long-term ambient stability.^67^ Recently, low-temperature solution-processed CuCrO_2_ nanocrystals have been demonstrated to be an effective HTL in inverted PSCs leading to 19.0% PCE with a significant improvement of device photostability.^68^

### Chemical doping

**Fig. 4:**
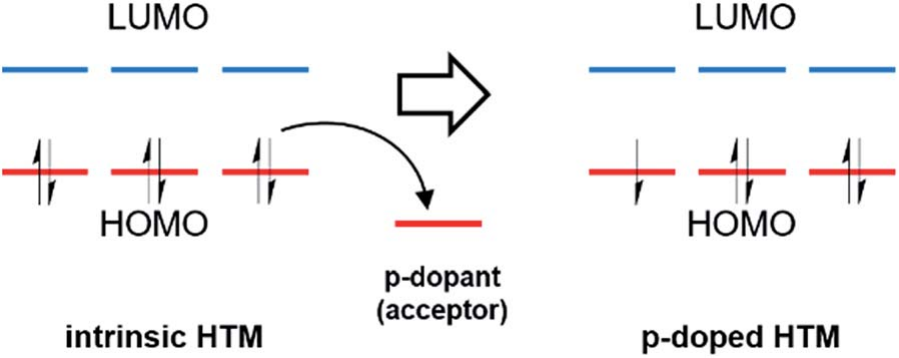
Schematic illustration of the molecular p-type doping mechanism, where the dopant is an acceptor.

Chemical doping is an important method in organic electronics to enhance device efficiency by controlling the energy levels, increasing the conductivity, and reducing ohmic losses in charge transport layers and injection barriers at the interface with the electrodes.^69^,^70^ The basic principle of p-doping in organic semiconductors is that additional mobile charge carriers are generated by electron acceptors, which remove electrons from the HOMO to generate holes of an intrinsic HTM (Fig. 4).^71^,^72^

The introduction of additional charge carriers leads to an increased charge carrier density in the organic semiconductor, resulting in higher conductivity. The key requirement for an efficient doping process is the suitable electron affinity of the dopant with respect to the energy levels of an organic semiconductor.

**Fig. 5:**
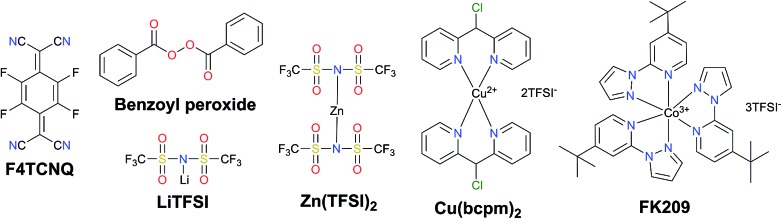
Chemical structures of different p-type dopants for HTMs employed in PSCs.

**Fig. 6:**
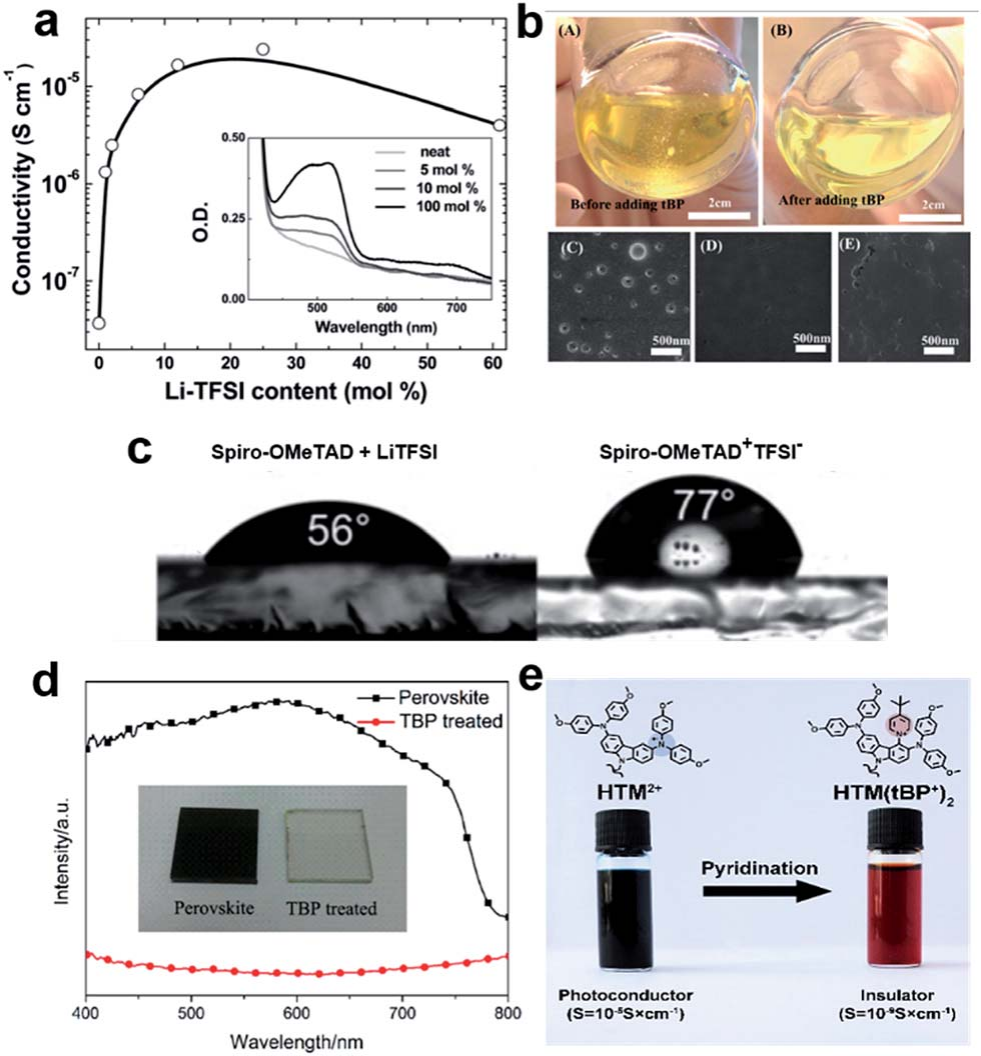
(a) Effective spiro-OMeTAD conductivity and UV-vis absorption spectra (inset) as a function of the LiTFSI content. These films were left for 78 hours in air before measurement. The solid-line is simply to aid the eye. Reprinted with permission from ref. 86. (b) Photographs of a HTL solution used for spin coating. (A) Before adding *t*BP and (B) after adding *t*BP. Top-view SEM images of the freshly prepared HTL (C) without *t*BP, (D) with *t*BP, and (E) with *t*BP after overnight vacuum treatment (10^–4^ Pa). Reprinted with permission from ref. 85. (c) Contact angle measurements of water droplets on films of spiro-OMeTAD doped with LiTFSI and spiro-OMeTAD^+^TFSI^–^ salt. Reprinted with permission from ref. 88. (d) UV-vis spectra of a TiO_2_/perovskite film and *t*BP-treated film, a photo of the films is shown in the inset. Reprinted with permission from ref. 90. (e) Schematic representation of the chemical reaction of the hole transporting material with *t*BP which occurs during the doping procedure resulting in new products with different properties. Reprinted with permission from ref. 91.

Traditionally, the hole transporting layer of PSCs is heavily doped with p-type dopants to provide the necessary electrical conductivity for the state-of-the-art spiro-OMeTAD and other wide bandgap HTMs. Numerous p-type dopants have been developed and realized including 2,3,5,6-tetrafluoro-7,7,8,8-tetracyanoquinodimethane (F4TCNQ),^73^–^75^ benzoyl peroxide,^76^ and copper(ii)^77^ and cobalt(iii)^78^,^79^ complexes (Fig. 5). To date, the combination of second generation cobalt complex-based tris(2-(1*H*-pyrazol-1-yl)-4-*tert*-butylpyridine)cobalt(iii) tri[bis(trifluoromethane)sulfonimide] (FK209)^80^ and lithium bis(trifluoromethanesulfonyl)imide (LiTFSI) with an additive of 4-*tert*-butylpyridine (*t*BP) as the morphology controller is routinely employed to improve the conductivity of HTMs (Fig. 6a and b).^81^–^86^ Very recently, incorporation of ZnTFSI_2_ as a dopant showed increased hole mobility and long-term operational stability.^87^

On the other hand, the use of dopants is problematic and can cause several stability problems due to the sophisticated oxidation procedure and undesired ion migration. Furthermore, the hygroscopic nature of LiTFSI makes the HTL highly hydrophilic showing a tendency towards chemical degradation, negatively influencing the stability of the entire device (Fig. 6c).^88^,^89^ Moreover, *t*BP has been revealed to interact with perovskite and may dissolve it, inducing a corrosion phenomenon, and may even cause a chemical reaction with the HTM (Fig. 6d and e).^90^,^91^

### Molecular doped HTMs

It is worth noting that reports on the first novel hole transporting materials for perovskite solar cells started to be published in 2014, about a year after perovskite photovoltaic research began. One has to understand that the knowledge at that time was completely different from that of the present time in 2019, resulting in sluggish progress in the beginning. However, during a span of five years, around 400 HTM-based articles were reported, including inorganic and organic molecular and polymeric materials. It would be futile to list all the compounds individually because new ones are being published each month and only a fraction shows a promising performance of at least 18%. Therefore, in this review, only the flagship HTMs will be reviewed, which have exhibited the highest performance so far. Summarized photovoltaic parameters of champion devices are listed in Table 1.

**Table 1:** Photovoltaic performance of the perovskite devices employed with the best performing doped small molecule-based HTMs sorted by PCE of the champion device

ID	*J*_sc_ (mA cm^–2^)	*V*_oc_ (V)	FF	PCE (%)	Ref. PCE^*a*^ (%)	Lit. ref.
**KR216**	22.3	1.023	0.77	17.8	18.4	108
**V950**	22.5	1.07	0.74	17.8	18.6	120
**LD29**	22.02	1.11	0.75	18.04	18.25	129
**PPyra-TXA** ^*d*^	20.6	1.10	0.797	18.06	16.15	99
**TTPA-DBQT**	22.23	1.09	0.744	18.08	17.96	148
**TTPA-BDT**	23.0	1.07	0.736	18.1	17.7	145
**ATT-OMe**	21.75	1.07	0.781	18.13	17.80	146
**H16**	23.28	1.108	0.704	18.16	18.27	131
**BTT-5**	22.50	1.10	0.733	18.17	18.9	143
**BTF-1**	21.31	1.106	0.772	18.2	18.0	144
**BTT-3**	21.9	1.065	0.767	18.2	18.1	142
**dly-2**	23.24	1.039	0.755	18.23	19.59	115
**V1050** ^*d*^	22.0	1.05	0.795	18.3	18.9	119
**KR131**	20.7	1.145	0.77	18.3	17.9	122
**CZ-TA** ^*d*^	21.66	1.044	0.81	18.32	18.28	125
**HL-2**	21.30	1.09	0.79	18.34	18.77	126
**V885**	22.09	1.107	0.757	18.45	18.79	118
**Dispiro-OBuTAD** ^*d*^	22.79	1.08	0.75	18.46	17.82	104
**F33** ^*d*^	21.01	1.11	0.79	18.48	16.26	114
**EH44** ^*e*^	21.71	1.091	0.779	18.5	19.6	128
**BTSe-1**	21.88	1.065	0.795	18.5	18.0	144
**JY6** ^*d*^	21.39	1.066	0.81	18.54	16.24	113
**Spiro-I**	24.67	1.06	0.71	18.57	19.17	102
**V1056**	22.1	1.07	0.79	18.7	20.2	121
**TTA3**	22.18	1.153	0.733	18.76	18.77	147
**NDT** ^*d*^	23.47	1.11	0.74	18.78	18.05	130
**CuPc**	22.6	1.07	0.775	18.8	20.5	141
**P_Zn_-2FTPA** ^*d*^	22.21	1.125	0.754	18.85	19.23	139
**V911**	22.4	1.103	0.763	18.86	18.79	118
**SGT-405**	22.93	1.046	0.786	18.87	17.71	111
**X36**	23.7	1.06	0.76	18.9	18.34	100
**V885**	22.43	1.104	0.764	18.92	18.79	118
**BTT-4**	23.04	1.090	0.753	18.97	18.9	143
**Si-OMeTPA** ^*b*^	23.08	1.07	0.771	19.06	17.47	107
**Z30**	23.53	1.114	0.73	19.17	19.66	134
**KR374**	23.15	1.092	0.762	19.26	19.54	110
**Py-OMe**	22.82	1.11	0.761	19.28	18.57	116
**POZ10**	23.1	1.1	0.762	19.4	19.7	136
**AZ2**	22.94	1.14	0.746	19.4	19.1	132
**DDOF**	22.37	1.101	0.79	19.4	18.8	105
**WT3**	22.6	1.095	0.785	19.44	18.62	140
**V859**	23.3	1.120	0.75	19.47	18.25	137
**HTM-FX** ^*d*^	—	—	—	19.5	20.4	94
**XPP** ^*c*^ ^,^ ^*d*^	23.18	1.12	0.75	19.5	16.5	97
**P1**	23.184	1.118	0.761	19.8	19.9	138
**H11**	24.2	1.150	0.71	19.8	18.9	109
**X59**	23.4	1.13	0.73	19.8	20.8	96
**IDIDF**	23.6	1.06	0.792	19.8	16.8	124
**X60**	24.2	1.14	0.71	19.84	—	93
**V862**	22.5	1.139	0.77	19.96	18.25	137
**Z26**	23.59	1.132	0.75	20.1	20.6	133
**SCZF-5** ^*d*^	24.40	1.11	0.74	20.1	19.11	103
**V1091** ^*d*^	22.5	1.11	0.81	20.2	20.2	121
**FDT**	22.7	1.148	0.76	20.2	19.7	98
**G2** ^*d*^	23.52	1.13	0.76	20.2	19.25	106
**X26**	24.3	1.11	0.75	20.2	18.34	100
**EDOT-Amide-TPA** ^*d*^	22.7	1.16	0.77	20.3	19.7	135
**TAT-*^t^*BuSty** ^*d*^	22.1	1.15	0.8	20.3	20.3	123
**OMe-TATPyr** ^*d*^	23.3	1.10	0.806	20.6	18.4	117
**HTM-FX'** ^*d*^	—	—	—	20.8	20.4	94
**X55**	23.4	1.15	0.77	20.8	18.8	101
**DM**	24.91	1.144	0.812	23.2	21.3	95

^*a*^Spiro-OMeTAD or PTAA was used as the reference in most of the cases.

^*b*^p–i–n device architecture.

^*c*^ *t*BP-free composition.

^*d*^Planar device architecture.

^*e*^LiTFSI-free composition.

As mentioned, spiro-OMeTAD is still dominating the field and despite its high price (∼350 $ per g) it is routinely employed as a highly efficient reference material for research. This is mainly because it is widely available and well-studied since it was commercialized decades ago. However, the potential for practical applications of spiro-OMeTAD is hardly possible. Therefore, huge research interest directed at new HTM candidates has been devoted to finding an ideal HTM which would be easily scalable at a reasonable cost as well as for a better understanding of the relationship between the HTM structure and PSC performance.

**Fig. 7:**
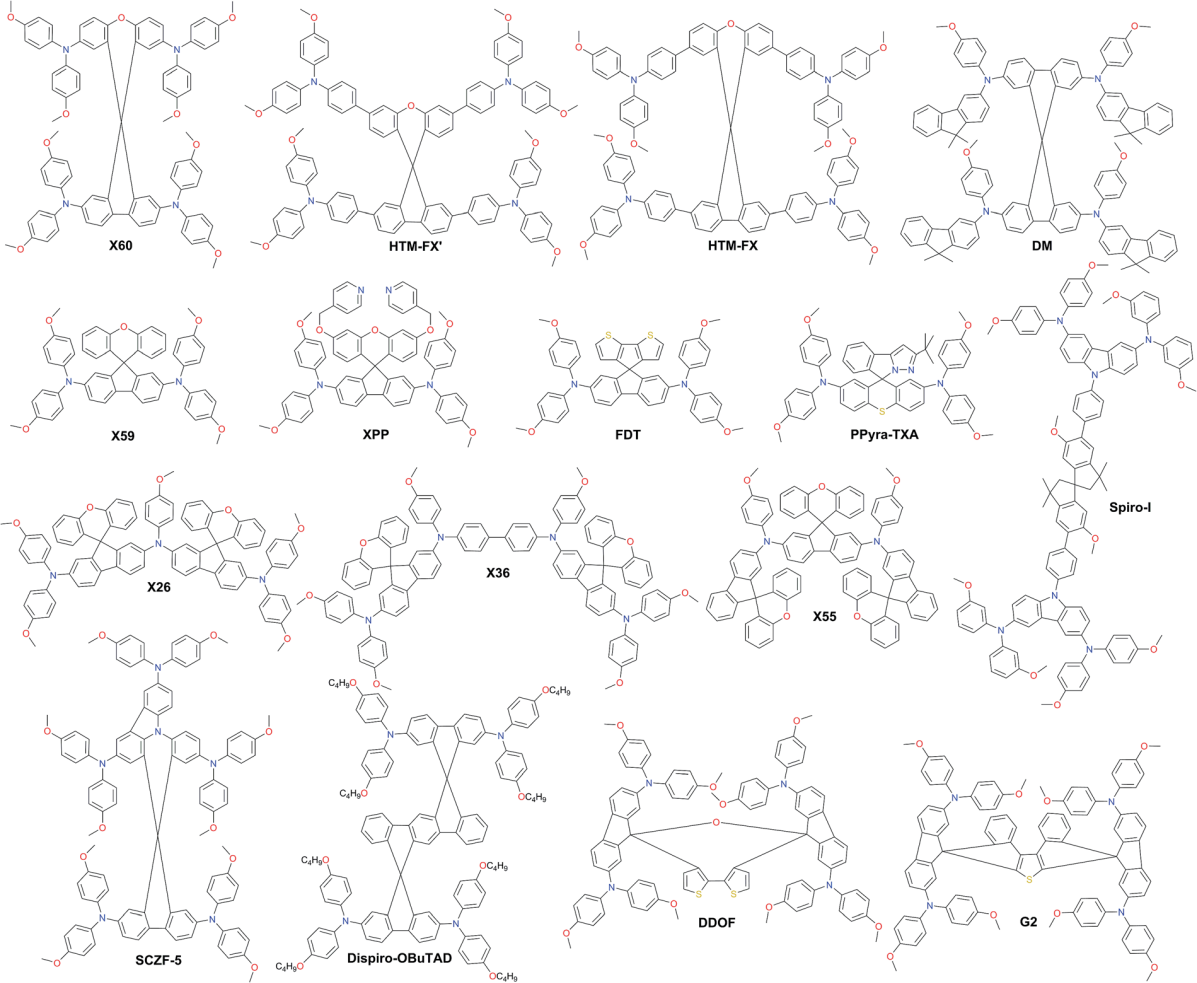
Chemical structures of best-performing spiro-based HTMs.

**Fig. 8:**
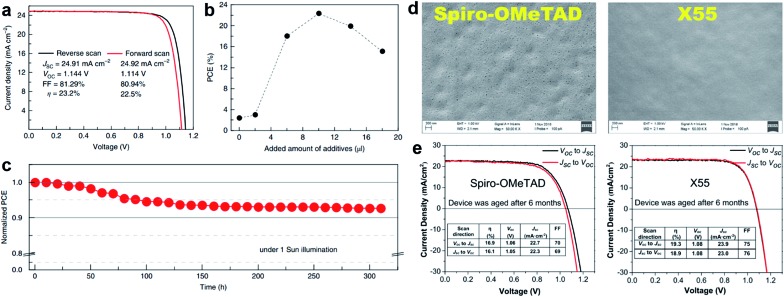
(a) *J*–*V* curves measured by reverse and forward scans of the best cell using **DM** with active area of 0.0939 cm^2^; (b) relationship between the power conversion efficiency and the amount of additive added to **DM**. The added amount of additives indicates the sum amount of LiTFSI solution in acetonitrile (340 mg ml^–1^) and pure *t*BP with a 1 : 1 volume ratio; (c) evolution of normalized power conversion efficiency obtained from a **DM**-based device with encapsulation under maximum power point tracking and continuous light irradiation (AM 1.5 G, 100 mW cm^–2^, white LED) at 25 °C. Initial efficiency is 20.8% with a *J*_SC_ of 24.3 mA cm^–2^, a *V*_OC_ of 1.11 V and a FF of 76.8%, which were measured from the *J*–*V* curves. Reprinted with permission from ref. 95. (d) Scanning electron microscopy (SEM) images of the HTM layer (top view); (e) *J*–*V* characteristics of spiro-OMeTAD and **X55** based PSCs under different scan conditions after aging for 6 months. Reprinted with permission from ref. 101.

As a result of the success of spiro-OMeTAD, many research groups have focused on spiro-type compounds and tried to improve the PCE with slight structural modifications.^92^ Some representative examples of these modifications are shown in Fig. 7. Xu *et al.* have reported **X60**, where the spirobifluorene central core is replaced by spiro[fluorene-9,9′-xanthene] (SFX), which is synthesized by a one-pot approach condensing 4-bromophenol and 2,7-dibromo-9-fluorenone as the starting materials. This allowed reduction of the price of the final product by thirty times compared with that of spiro-OMeTAD. Moreover, the insertion of oxygen bridge into the structure leads to improved hole mobility and a device performance of 19.8%.^93^ Very recently, Chiykowski *et al.* judiciously studied redox and thermal properties of SFX-based HTMs bearing TPA groups. It was determined that TPA groups positioned about the conjugated fluorene moiety increase the free energy change for hole-extraction from the perovskite layer, while TPAs about the xanthene unit govern the *T*_g_ values and hole mobility. 4-fold **HTM-FX** and **HTM-FX’** exhibited PCEs of 19.5% and 20.8%, respectively.^94^ Recently, the fluorene-terminated spiro-OMeTAD alternative **DM** has been reported to have achieved a record efficiency of 23.2% so far. Such a high PCE is achieved by the careful optimization of different amounts of additives (LiTFSI and *t*BP) in the HTL. Without additives, or with only small amounts of additives, the PCE of the device is very low because the conductivity of **DM** is not high enough. An encapsulated device using **DM** retained 92.6% of its initial PCE value after 310 h under continuous illumination conditions demonstrating its enhanced thermal stability compared with that of spiro-OMeTAD (Fig. 8a–c).^95^

Two-fold dimethoxydiphenylamine-substituted **X59** was reported a few months later, with an identical device performance to that of its predecessor **X60** and almost no hysteretic behavior,^96^ showing that diphenylamine units on xanthene do not play a major role; therefore it was further functionalized with covalently linked differently substituted pyridine groups. This allowed realization of *t*BP-free PSCs with improved stability compared with that of spiro-OMeTAD-based solar cells that contained *t*BP as an additive.^97^*Para*-position substituted pyridine-based **XPP** showed the highest performance of 19.5% among the series, due to the fact that both nitrogen atoms in **XPP** can easily form strong chemical bonds with Pb atoms, while the *ortho*- and *meta*-anchored molecules can only contribute one nitrogen atom for binding to Pb. However, the pyridine anchors slightly lowered the performance when compared with **X59**. Another interesting strategy to improve hole transfer at the HTM/perovskite interface was demonstrated employing **FDT** as the HTM, where the cyclopenta[2,1-*b*:3,4-*b*′]dithiophene moiety acts as an anchor to reveal an additional thiophene–iodine interaction.^98^ This led to one of the highest reported device performances so far of 20.2% with a *J*_SC_ of 22.4 mA cm^–2^, *V*_OC_ of 1.15 V and FF of 0.76. C–N linked spiro structures were reported in order to investigate unsymmetrical spiro configurations, consisting of phenylpyrazole and different heteroaromatic fragments.^99^ S-bridged **PPyra-TXA** showed the highest PCE of 18.06% among the reported series.

Zhang *et al.* further studied X-series molecules based on the SFX core. Very recently, they reported **X26** and **X36** molecules, which could be considered as differently connected dimers of **X59**. Connection through one of the nitrogen atoms in the diphenylamine unit led to **X26**, while connecting through one of the phenyl rings in diphenylamine yielded **X36**. The PSCs based on **X26** showed a remarkable power conversion efficiency of 20.2% in comparison to the 18.9% for **X36**-based devices, respectively. It was found that **X26** forms a homogeneous capping layer, with efficient interfacial hole transfer and fast charge collection, leading to the improved *V*_OC_ and *J*_SC_ of the device, when compared with **X36**.^100^ Furthermore, replacing one of phenyl rings in both diphenylamine arms with the SFX core gave the trimer **X55** with excellent 3D structured configuration.^101^ PSC devices with **X55** as the HTM showed an impressive PCE of 20.8%, with a *J*_SC_ of 23.4 mA cm^–2^, *V*_OC_ of 1.15 V and FF of 0.77. Correspondingly, the efficient charge collection in **X55**-based devices can be related to fast hole transport and interfacial hole transfer, which also explains their high FF and *J*_SC_. After aging for 6 months, the efficiency of the **X55**-based devices decreased by only 7% (from 20.8% to 19.3%), whereas the efficiency of the spiro-OMeTAD-based solar cells decreased by 10% (from 18.8% to 16.9%). The high uniformity and homogeneity of the **X55** film coverage on top of the perovskite material is a possible explanation for the good stability results (Fig. 8d and e). Remarkable performance and simplified synthetic schemes make **X60** and **X55** very promising HTM candidates for large-scale industrial production. A spirobisindane core was utilized to prepare **Spiro-I** in order to decrease the molecular symmetry of the spiro unit. This feature effectively suppresses the crystallization trend of **Spiro-I** resulting in uniform pinhole-free films with high morphological stability and a performance of 18.57% PCE.^102^

Very recently, Zhu and co-workers reported a novel design incorporating carbazole into the spiro core. A carbazole-based single-spiro-HTM named **SCZF-5** achieved a PCE of 20.1%.^103^ Another example, **Dispiro-OBuTAD** based on rigid ladder-type structured dispiro[9*H*-fluorene-9,6′(12′*H*)-indeno[1,2-*b*]fluorene-12′,9′′-[9*H*]fluorene] with tetrakis[*N*,*N*-di(4-butoxyphenyl) amino] groups as peripheral substituents showed great steric hindrance with reduced molecular symmetry and a PCE of 18.46%.^104^ A 3D structured dispiro-oxepine derivative **DDOF** was designed using a 3-step synthetic route and showed a PCE of 19.4% with improved long-term stability compared with spiro-OMeTAD.^105^ A similar thiophene-based dispiro functionality **G2** has been reported to achieve an efficiency of 20.2%.^106^

Very recently, the silicon core X-shaped molecule **Si-OMeTPA** has been reported to achieve a PCE of 19%.^107^ This novel design also offers a facile two-step synthesis process. The 9,9′-bifluorenylidene-based hole-transporting material **KR216**, obtained by a straightforward two-step synthetic route was reported, and it mimics the synthetically challenging 9,9′-spirobifluorene moiety.^108^ The estimated price of **KR216** is around 50 times lower than that of commercial spiro-OMeTAD, and it yields a comparable power conversion efficiency of 17.8%. A higher device performance of 19.8% was achieved employing another spiro-OMeTAD analog **H11**, where the double bond linkage between the two planar fluorene halves is replaced by a single C–C bond.^109^ The impact of different atom incorporation into the **KR216** structure has been studied further. Replacement of the oxygen atom with sulfur showed a significantly improved hole-drift mobility leading to enhanced photovoltaic performance and reduced hysteresis most likely owing to the improved interface between the perovskite and HTM caused by stronger Pb–S interaction, whereas oxidation of sulfur to sulfone has a negative effect on the device performance. S-bridged **KR374** showed a promising performance of 19.2%.^110^ Lu *et al.* reported carbazole-based starburst HTMs *via* tuning the carbazole substitution position from 2,7- to 3,6-, respectively. 3,6-substituted **SGT-405** showed a slightly improved device performance of 18.9%, compared with that of 18% in the case of 2,7-substitution.^111^

**Fig. 9:**
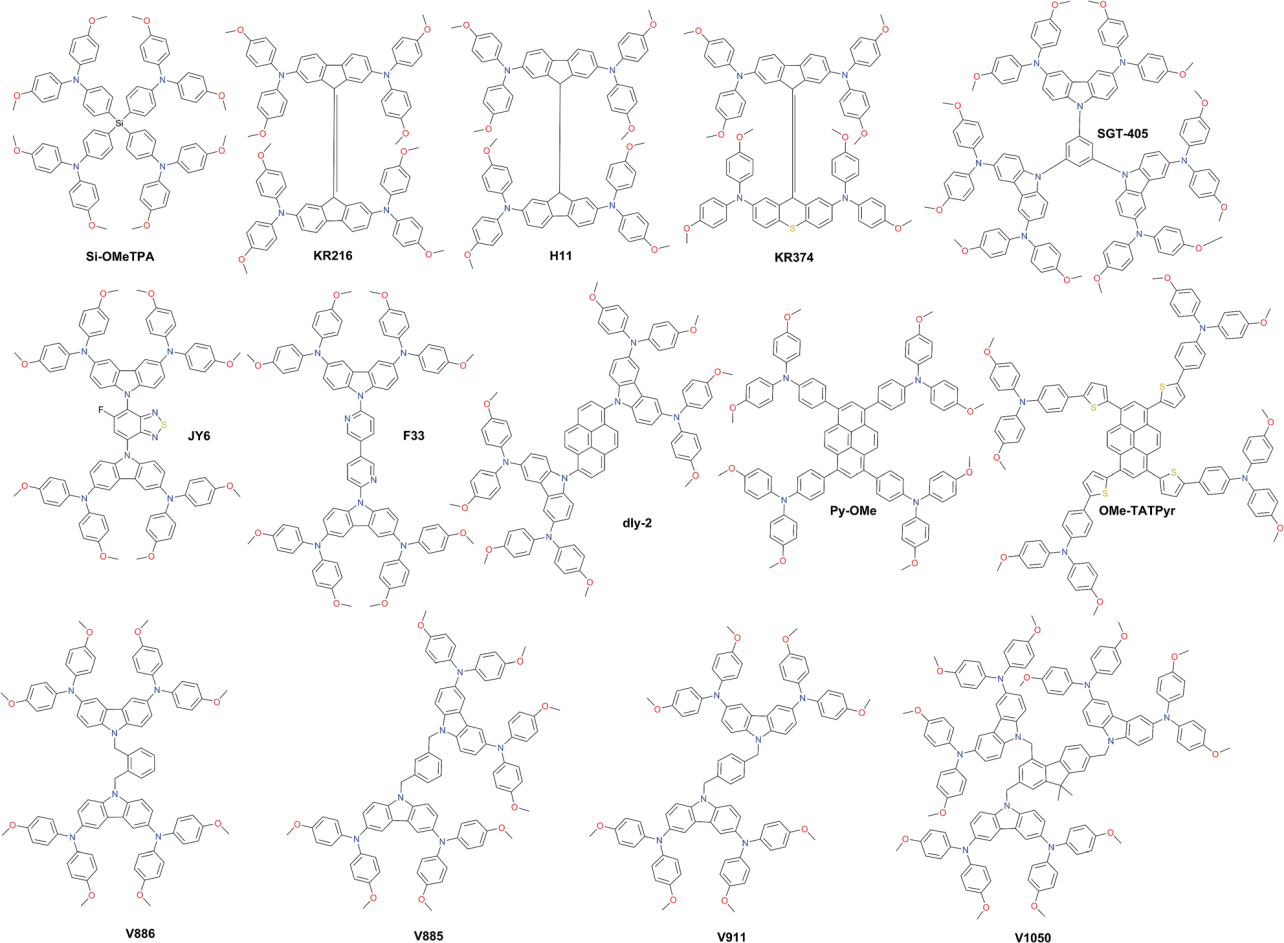
Chemical structures of highly efficient hole transporting materials employed in perovskite solar cells.

Carbazole derivatives have been intensively studied since they have interesting features such as the low-cost of the 9*H*-carbazole starting material, good chemical and environmental stability provided by the fully aromatic unit, and easy incorporation of a wide variety of functional groups into the nitrogen atom that allows better solubility and fine tuning of the electronic and optical properties.^112^ 3,6-Dimethoxydiphenylamine-substituted carbazoles as donor units at the periphery were widely used to tune the HOMO level of the final molecule. Some representative examples of such systems are shown in Fig. 9. Wu and co-workers reported benzothiadiazole (BT)-based HTM series.^113^ The monofluorinated BT in **JY6** increases its hole mobility, hole extraction, and hole transporting ability, and thus significantly enhances the photovoltaic performance (*η* = 18.5%) compared with non-fluorinated (*η* = 16.9%) and difluorinated (*η* = 15.7%) analogs. The same photovoltaic performance was recently obtained with **F33**, where BT was replaced by 3,3′-bipyridine.^114^

Li *et al.* functionalized a two-dimensional pyrene core with two- and four-fold electron-rich substitutions.^115^ 18.2% PCE in planar PSCs has been achieved by employing disubstituted **dly-2**, which is slightly higher in comparison with that in PSCs using tetrasubstituted derivatives. Very recently, another pyrene HTM **Py-OMe** comprising four triarylamine substituents was reported by Cui *et al.* to achieve a PCE of 19.28%.^116^ Bridging the latter with extra thiophene units yielded **OMe-TATPyr** and further improved the photovoltaic performance over 20%.^117^ A family of branched HTMs containing two diphenylamine-substituted carbazole fragments linked by a non-conjugated differently substituted methylenebenzene unit has been extensively studied by Getautis *et al.*^118^*ortho*-, *meta*-, and *para*-dimethylenebenzene linked isomers **V886**, **V885**, and **V911**, respectively, were investigated recently. The performance of **V885** and **V911** is on a par with that of spiro-OMeTAD, reaching 18.9%, while the introduction of additional branches or substitution of methoxy groups in place of methyl did not lead to higher efficiency. A slightly lower performance of 18.3% has been achieved with **V1050**, where the central benzene unit is replaced with fluorene.^119^ Owing to the simplicity of synthesis for the V-family molecules it holds great promise for practical application in PSCs.

**Fig. 10:**
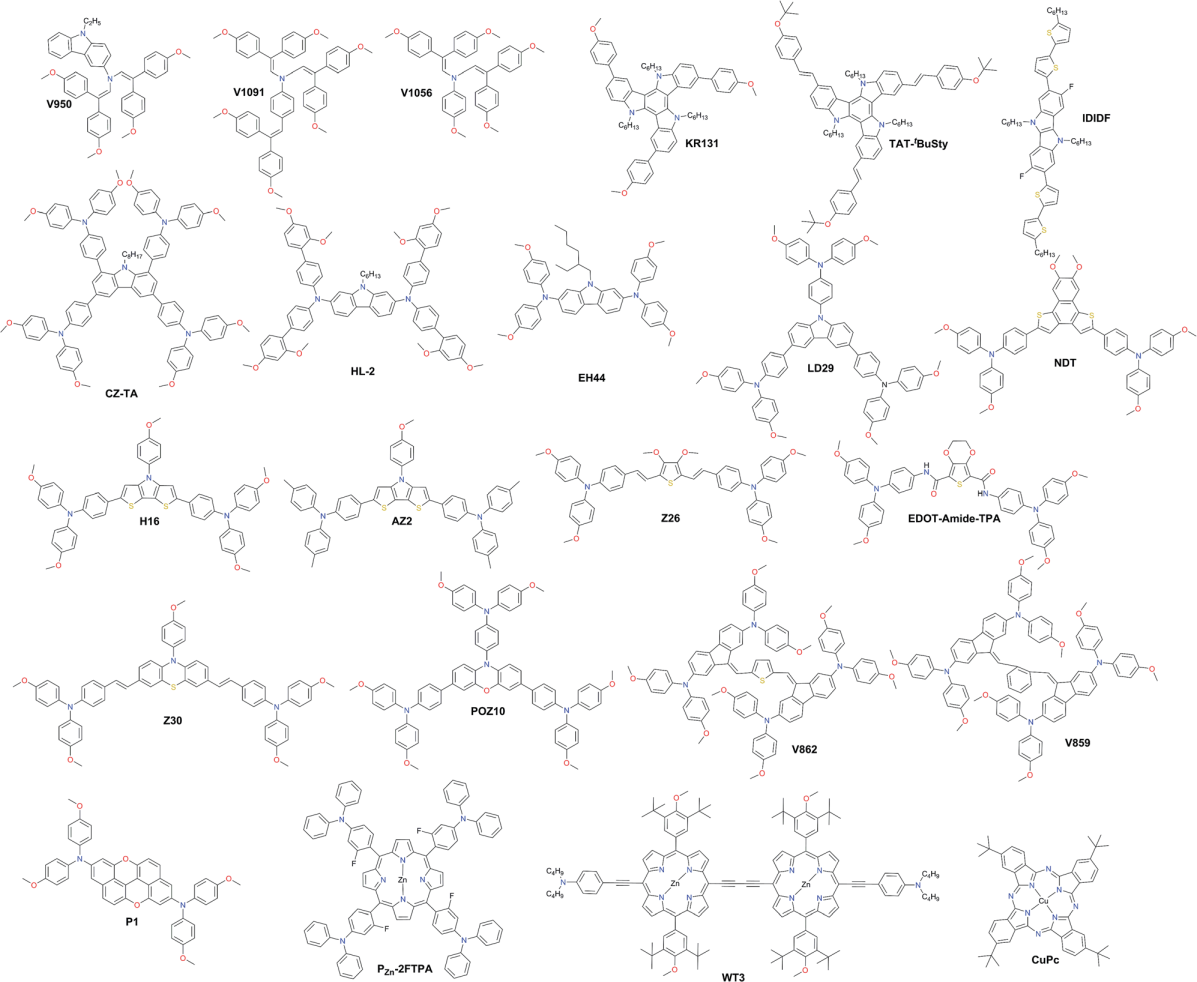
Chemical structures of hole transporting materials employed in high performance perovskite solar cells.

In Fig. 10, the most efficient carbazole-based HTMs are shown. The low-cost enamine **V950** was synthesized *via* an extremely simple route from commercially available and relatively inexpensive starting reagents, resulting in more than one order of magnitude lower cost of the final product compared to the commercial spiro-OMeTAD, and showed a PCE of 17.8%.^120^ Very recently, the PCE has been further improved with aniline-based enamines using the same straightforward synthetic protocol. Three diphenylethenyl moieties containing **V1091** showed an excellent hole mobility of 1.7 × 10^–2^ cm^2^ V^–1^ s^–1^ leading to a PCE of 20.2%, while **V1056** showed a slightly lower performance of 18.7%.^121^ In one of the earliest reports, 18.3% device performance was achieved employing triazatruxene-based **KR131**, which exhibited surface interaction with the perovskite material resulting in efficient hole injection from the valence band of perovskite into the HOMO of the HTM, outperforming the reference spiro-OMeTAD.^122^ Very recently, another triazatruxene HTM **TAT-*^t^*BuSty** was reported to achieve a PCE of 20.3%.^123^ Cho and co-workers reported a fluorinated indolo[3,2-*b*]indole-based derivative **IDIDF**.^124^ A planar π-conjugated backbone linked with a flexible alkyl chain enabled the formation of a molecular stacked arrangement with strong π–π interaction, leading to improved hole mobility and a PCE of 19.8%.

The carbazole-based examples **CZ-TA**^125^ and **HL-2** 126 showed an identical photovoltaic performance of 18.3%. From a structural point of view, both have long alkyl chains for solubility improvement, octyl and hexyl, respectively, whereas the peripheral donors are completely different. **CZ-TA** possesses 1,3,6,8-substituted *p*-dimethoxytriphenylamine units, while **HL-2** is 2,7-substituted with *o*,*p*-dimethoxyphenyl-extended diphenylamine units. Very recently, a significantly enhanced photovoltaic performance of 19.85% and device stability have been realized by adding 10 wt% of the sister molecule methylthio-substituted **CZ-STA** to **CZ-TA** as a blend HTL.^127^ A simple dimethoxydiphenylamine substituted *N*-ethylhexyl carbazole HTM **EH44** was recently reported to achieve 18.5% PCE with 1000 hour operational stability due to HTM oxidation by AgTFSI.^128^ The star-shaped carbazole derivative **LD29** has three OMe-TPA units. The simple design of **LD29** gave a performance of 18.04%.^129^

Fused thiophene rings are attractive candidates as building blocks for HTM synthesis due to better π-conjugation across the molecule; some of the most efficient examples are shown in Fig. 10. A naphtho[1,2-*b*:4,3-*b*′]dithiophene-based HTM, **NDT**, has been reported by Cui and co-workers and showed a PCE of 18.8%.^130^ Another similar example employing the dithieno[3,2-*b*:2′,3′-*d*]pyrrole derivative **H16** was reported to achieve a PCE over 18%.^131^ Replacing methoxy groups on TPA with methyls improved the molecular stacking and led to a significantly higher performance of 19.4% achieved with **AZ2** by Zheng and co-workers.^132^ Very recently, Zhang and co-workers reported two HTMs with the introduction of vinylene bridges between the central core and electron-rich triarylamine donating units through the Wittig reaction, denoted as **Z26** and **Z30**. Thiophene-based **Z26** exhibited an impressive PCE of 20.1%.^133^ Due to the introduction of double bonds, **Z26** presents a more homogeneous surface, higher hole mobility, and higher conductivity than its analog, where *p*-dimethoxytriphenylamine units are connected directly to the thiophene. The second one, **Z30** based on phenothiazine, showed a slightly lower performance of 19.2%.^134^ Petrus *et al.* introduced an amide-functionality in the backbone of HTMs to synthesize an **EDOT-Amide-TPA** material by a simple condensation reaction which showed a very high performance of 20.3%.^135^ Phenoxazine-based **POZ10** reported by Chen and co-workers showed a PCE of 19.4%.^136^ Other very interesting branched molecules **V862** and **V859**, bearing *p*-dimethoxydiphenylamine-substituted fluorene fragments, were reported by Malinauskas *et al.* and are presented in Fig. 10.^137^ They can be produced in a two-step procedure from cheap, commercially available starting reagents, and therefore are very promising for large-scale production. Furthermore, **V862** yielded a maximum PCE up to 20%, whereas **V859** showed a slightly lower value of 19.5%. A similar performance of 19.8% was obtained with the *peri*-xanthenoxanthene centered HTM **P1** reported by Xu *et al.*^138^

Porphyrin derivatives have been widely studied as hole transport layers due to their electron rich characteristics. Azmi *et al.* reported Zn porphyrin-based HTMs with conjugating fluorinated triphenylamine wings. The best among the series **P_Zn_-2FTPA** exhibited a PCE of 18.85%.^139^ Chiang *et al.* designed and synthesized the dimeric porphyrin material **WT3** with a suitable HOMO level and hole mobility leading to an excellent PCE of 19.44%.^140^ Phthalocyanine derivatives have also been successfully applied as HTMs, owing to their specific molecular ordering, thereby giving rise to a highly crystalline and robust stack with high hole mobility. To date, the most successful example of this class has been **CuPc**, which resulted in the highest PCE of 18.8% with outstanding long-term stability against thermal stress under an air atmosphere.^141^

**Fig. 11:**
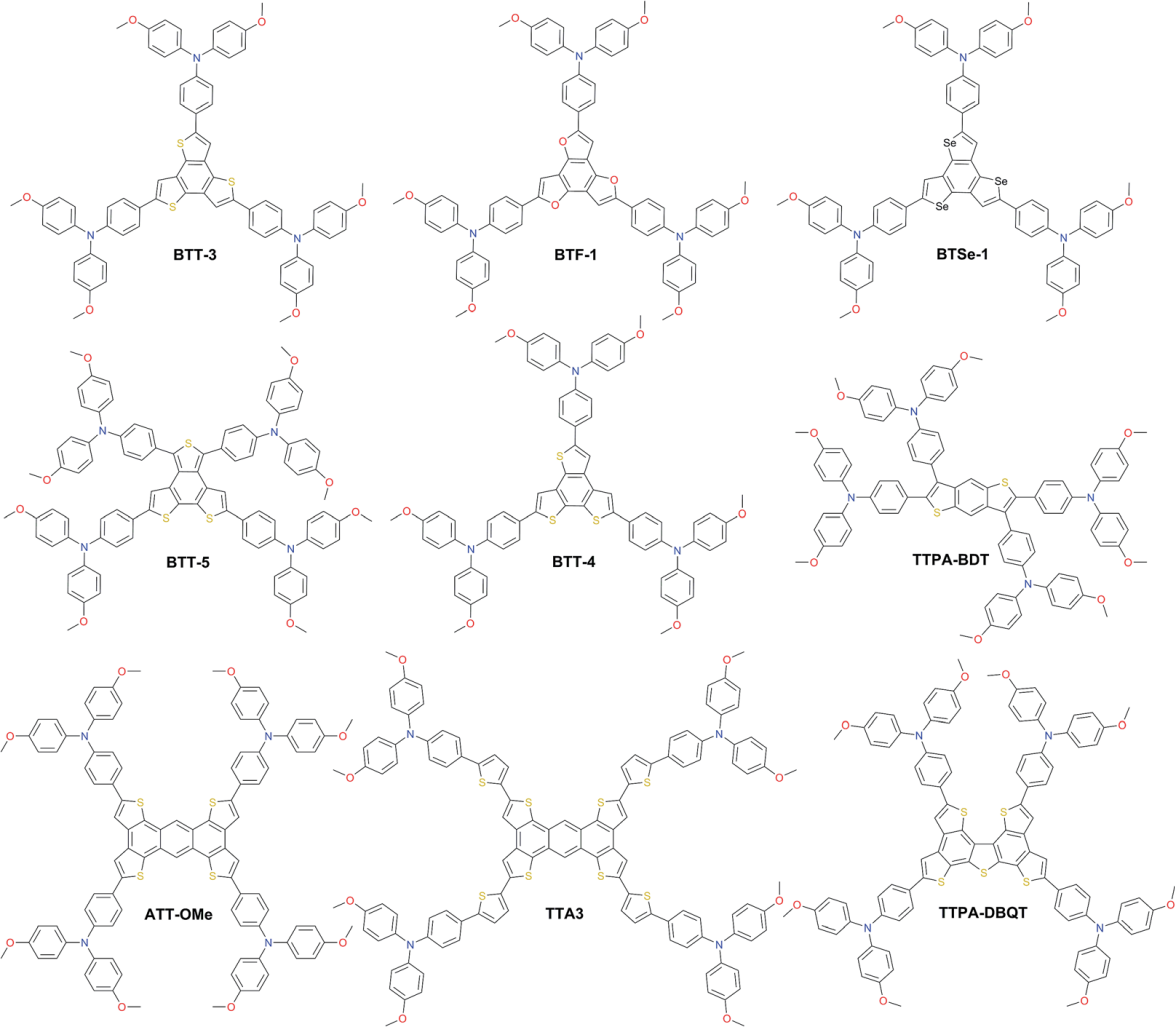
Chemical structures of the most efficient hole transporting materials based on benzodithiophene (BDT), benzotrithiophene (BTT), benzotrifuran (BTF), benzotriselenophene (BTSe), anthratetrathiophene (ATT), and dibenzoquinquethiophene (DBQT) cores.

HTMs based on the sulfur-rich benzotrithiophene (BTT) core were intensively studied as such fused systems are known to maximize the π-orbital overlap, inducing face-to-face π-stacking and facilitating charge transport through intermolecular hopping. Star-shaped **BTT-3** was first reported with an optimized HOMO level, showing an excellent band alignment with the perovskite, leading to a PCE of 18.2%.^142^ Encouraged by this result, Garcia-Benito *et al.* have further studied the impact of isomerism on BTT-based HTMs.^143^ It was found that *cis* arrangement of the sulfur atoms facing each other in the core of **BTT-4** may further benefit the interaction with the perovskite, leading to an impressive PCE of 19%, while the 4-fold isomeric form of **BTT-5** showed 18.2% PCE. Very recently, the heteroatom effect (N, O, and Se) was studied yielding benzotripyrrole (BTP), benzotrifuran (BTF), and benzotriselenophene (BTSe) central cores, respectively. From single-crystal diffraction, it was found that **BTF-1** shows tighter molecular π–π interactions than **BTT-3** and **BTSe-1**. However, the best PSC performance of 18.5% was achieved with **BTSe-1**, while **BTF-1** showed a slightly lower result of 18.2%.^144^ Benzodithiophene (BDT) and the more extended sulfur-rich core anthratetrathiophene (ATT) were also used as scaffolds for novel HTMs. Sandoval-Torrientes *et al.* reported **TTPA-BDT**^145^ molecule with four-fold substitution bringing the performance (*η* = 18.1%) close to that of BTT analogs. A similar PCE was achieved with devices prepared with the anthratetrathiophene-based **ATT-OMe**, which showed the highest efficiency of 18.13% among a series of different alkoxy length HTMs, showing that longer alkoxy chains are not beneficial.^146^ Introduction of extra thiophene rings between the ATT central core and triarylamine yielded **TTA3** and led to an increased PCE of 18.7%.^147^ Further engineering of the ATT scaffold led to dibenzoquinquethiophene (DBQT), where the central benzene ring is replaced with thiophene. However, the PCE of **TTPA-DBQT** HTM remained almost identical to that of **ATT-OMe** reaching 18.08% (Fig. 11).^148^

In conclusion, tremendous effort has been made to develop new alternative low-cost and efficient HTMs to replace spiro-OMeTAD, and organic small-molecule HTMs are the most widely developed group in PSCs owing to their synthetic variety, tuneable properties, high purity, and simple solution processing. However, although the performance of PSCs is dramatically enhanced, most of the reported high-efficiency doped HTMs suffer from rapid degradation and are limited by a very short operation lifetime of up to a few months.

### Molecular dopant-free HTMs

**Fig. 12:**
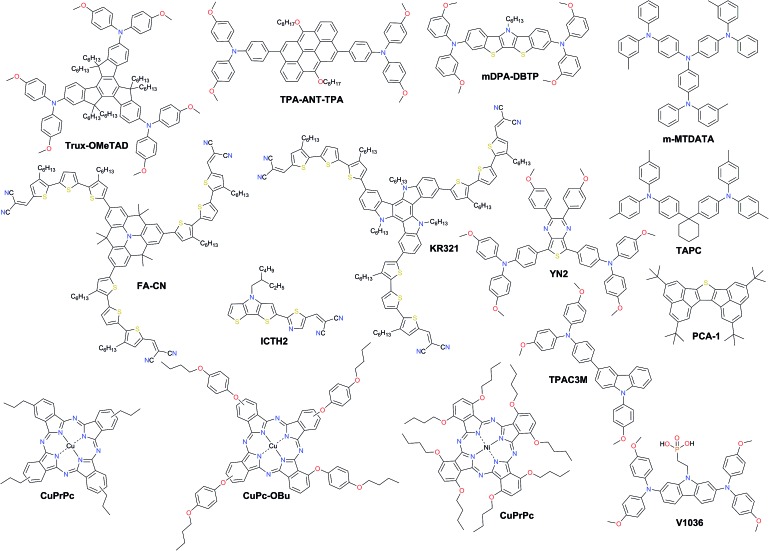
Chemical structures of the most efficient dopant-free hole transporting materials applied in PSCs.

A promising solution for stabilizing PSCs is the appropriate choice of dopant-free HTMs. However, the PCEs of pristine HTL devices consistently lie around 15% with only very few examples over 18% (Fig. 12 and Table 2). The long-term stability of PSCs is one of the critical issues that need to be addressed for their commercial application. Removing the dopants has already been demonstrated to be very effective towards improved device stability.

**Fig. 13:**
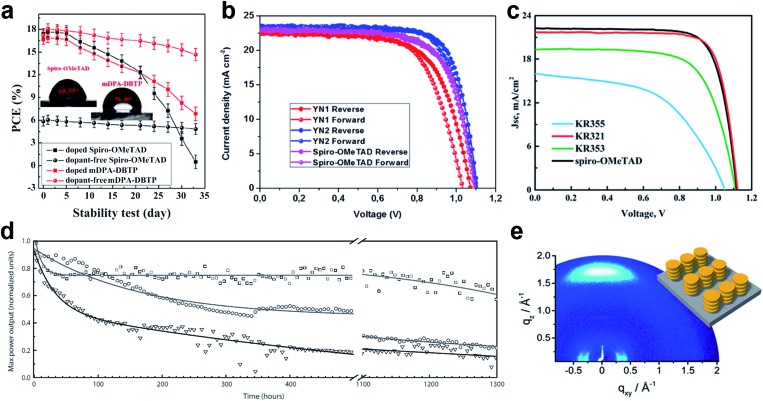
(a) Performance stability of unencapsulated PSCs based on ***m*DPA-DBTP** under ambient air storage (at a humidity level of 40 ± 5% and a temperature of 25 ± 3 °C). Reprinted with permission from ref. 151. (b) *J*–*V* curves of PSCs based on the **YN2** HTM. Reprinted with permission from ref. 156. (c) *J*–*V* curves of the dopant-free HTM **KR321** and doped spiro-OMeTAD as the reference. Reprinted with permission from ref. 157. (d) Maximum power point tracking of perovskite solar cells prepared in a single experiment, using undoped **FA-CN** (□), **TPA-CN** ( (◌), and doped spiro-OMeTAD (∇) as hole-transporting materials. The measurements were performed under UV-filtered simulated sunlight in an argon atmosphere. The initial power conversion efficiency was measured after storing the device under argon and in the dark for 24 h. Reprinted with permission from ), and doped spiro-OMeTAD (∇) as hole-transporting materials. The measurements were performed under UV-filtered simulated sunlight in an argon atmosphere. The initial power conversion efficiency was measured after storing the device under argon and in the dark for 24 h. Reprinted with permission from ref. 158. (e) GIWAXS pattern of the **KR321** film coated with tetrachloroethane on a silica wafer (schematic illustration of the molecular surface arrangement). Reprinted with permission from ref. 157.

Huang *et al.* presented a dopant-free truxene-based HTM decorated with three diarylamine groups and six hexyl side chains, adopting a planar, rigid, and fully conjugated molecular geometry. PSCs fabricated with a p–i–n architecture using **Trux-OMeTAD** showed a high PCE of 18.6% with minimal hysteresis.^149^ The anthanthrone dye based molecule **TPA-ANT-TPA** has been reported by Pham *et al.* to achieve 17.5% PCE using no regular doping.^150^ Hazmi *et al.* reported dopant-free molecules based on dibenzothienopyrrole combined with diphenylamine donors tuned with differently positioned methoxy groups.^151^*meta*-substituted ***m*DPA-DBTP** showed the highest performance among the series achieving a PCE over 18%. Notably, devices based on dopant-free ***m*DPA-DBTP** retained 81% of its original PCE after 33 days of storage, during which the doped spiro-OMeTAD devices lost their PCE completely as shown in Fig. 13a. ***m*-MTDATA** has been reported to reach a PCE as high as 18.12% among simple solution-processed inverted PSCs with a *J*_SC_ of 22.50 mA cm^–2^, *V*_OC_ of 1035 mV and FF of 0.778. Furthermore, ***m*-MTDATA** based devices without any encapsulation showed significantly better stability than the PEDOT:PSS based devices after 1000 h exposure to an ambient environment with a humidity of 30 RH%.^152^ Another commercial example **TAPC** has been demonstrated to be an efficient HTM in p–i–n configuration PSCs. By adopting **TAPC** as the HTL, the best device achieved a PCE of 18.80% with minor hysteresis and increased stability without any encapsulation.^153^ Li with co-workers presented a simple polycyclic 2,5,9,12-tetra(*tert*-butyl)diacenaphtho[1,2-*b*:1′,2′-*d*]thiophene dopant-free HTM denoted as **PCA-1** with a PCE of 18.17%.^154^ Simple design of a **TPAC3M** dopant-free HTM has been reported by Park *et al.*^155^ Fabricated p–i–n inverse type planar PSCs with the configuration ITO/**TPAC3M**/CH_3_NH_3_PbI_3_/PCBM/ZnO/Al exhibited 17.54% PCE.

Donor–π-bridge–acceptor (D–π–A) type dopant-free HTMs have great potential to show high charge carrier mobility due to strong dipolar intermolecular interactions, minimizing ohmic losses of the contact. Moreover, they combine the advantages of both small molecules, like well-defined structures, and polymers, like good thermal, electrochemical and photochemical stability, together with high solubility and suitable wetting on the perovskite. Typically, they are composed of a planar central unit inducing the formation of a molecular stacked arrangement due to strong π–π interaction linked to a strong accepting unit and combined with flexible alkyl chains leading to improved hole mobility.

Recently, a D–π–A-type HTM coded as **FA-CN** was reported, incorporating a fused quinolizino acridine donor with alkyl-substituted terthiophenes and malononitriles as acceptors. Optimized devices with dopant-free **FA-CN** showed an impressive PCE of 18.9% with superior long-term stability under full illumination for 1300 h (Fig. 13d).^158^ Changing the quinolizino acridine central core to triazatruxene yielded the **KR321** HTM with a slightly improved performance of 19% due to the highly ordered characteristic face-on organization leading to increased vertical charge carrier transport (Fig. 13c and e).^157^ Bharath *et al.* reported the D–π–A type **ICTH2** HTM based on electron-donating dithieno[3,2-*b*:2′,3′-*d*]pyrrole and electron-accepting dicyanovinylene moieties imparting high intramolecular charge transfer nature and eliminating the need for additives or dopants during the fabrication of solar cell devices.^159^ PSCs with the **ICTH2** HTM showed a PCE of 18.75% with an active area of 2.04 cm^2^. Very recently, the D–A–D type HTM **YN2** featuring triphenylamine as the donor along with a thienopyrazine unit as the acceptor has been presented by Xu *et al.* The **YN2**-based PSC affords an impressive PCE value of 19.27% with a *V*_OC_ of 1.11 V, a *J*_SC_ of 23.15 mA cm^–2^ and a FF of 0.75 as shown in Fig. 13b.^156^

Another interesting class of dopant-free HTMs is phthalocyanines (Pcs). The optical and electronic properties of these macrocycles can be easily adjusted by modifying the peripheral and non-peripheral substituents and the metal center. Pcs have unique properties, such as ease of synthesis related to low production cost, relatively high hole mobility, and high hydrophobicity, as well as excellent chemical, thermal, and photo stability. So far, the highest performance using dopant-free layers in this class has been achieved by metal phthalocyanines, such as copper(ii) phthalocyanines (CuPcs) and nickel(ii) phthalocyanines (NiPcs). Liu *et al.* reported copper(ii) Pc with tetrapropyl side chain substitution denoted as **CuPrPc**.^160^ A comprehensive study by GIXRD and AFM confirmed that the surface of the **CuPrPc** film grown on perovskite has low surface roughness, high quality crystallization and face-on molecular orientation enhancing hole transport leading to a PCE of 17.8%. Moreover, it has a good coverage area and high hydrophobicity, improving the stability of PSC devices. Jiang *et al.* found that a small structural change from butyl groups to butoxy groups in the substituents of the Pc rings significantly influences the molecular ordering and effectively improves the hole mobility and solar cell performance. The optimized PSCs employing **CuPc-OBu** as the dopant-free HTM afforded a PCE of 17.6%.^161^ In addition, PSC devices containing the dopant-free **CuPc-OBu** HTM also exhibited excellent stability when stored without encapsulation under ambient conditions with a RH of 85% relative to those containing doped spiro-OMeTAD, mainly due to the high moisture resistivity of the **CuPc-OBu** interlayer. Chen *et al.* reported that solution-processable nickel phthalocyanine (NiPc) abbreviated **NiPc-(OBu)_8_** and vanadium (V) oxide (V_2_O_5_) are successfully incorporated into structured mesoporous PSCs showing a PCE of up to 18.3%.^162^

Magomedov *et al.* have demonstrated a hole-transporting self-assembled monolayer (SAM) as the dopant-free hole-selective contact in p–i–n PSCs.^163^ SAM formation is achieved by simply immersing the substrate into a solution of a novel molecule **V1036** that binds to the indium tin oxide surface due to its phosphonic anchoring group. A PCE of up to 17.8% with an average fill factor close to 80% and undetectable parasitic absorption was demonstrated. A slightly lower PCE of 17.3% was achieved by another SAM-forming molecule **MC-43** having a carboxylic anchor as proposed by Yalcin *et al.*^164^ Further gains in efficiency can be expected upon SAM optimization by means of molecular and compositional engineering.

**Table 2:** Photovoltaic performance of the perovskite devices employed with best performing dopant-free small molecule-based HTMs sorted by PCE of the champion device

ID	*J*_sc_ (mA cm^–2^)	*V*_oc_ (V)	FF	PCE (%)	Ref. PCE^*a*^ (%)	Lit. ref.
**TPA-ANT-TPA**	21.07	1.03	0.796	17.5	16.8	150
**TPAC3M** ^*b*^	22.79	1.00	0.78	17.54	12.6	155
**CuPc-OBu**	22.8	1.06	0.73	17.6	18.8	161
**CuPrPc** ^*c*^	23.2	1.01	0.76	17.8	17.5	160
**V1036** ^*b*^	21.9	1.09	0.81	17.8	19.2	163
**mDPA-DBTP** ^*c*^	21.13	1.12	0.76	18.09	17.82	151
***m*-MTDATA** ^*b*^	22.50	1.035	0.78	18.12	13.44	152
**PCA-1** ^*c*^	22.30	1.062	0.767	18.17	18.30	154
**NiPc-(OBu)_8_**	23.1	1.08	0.734	18.3	19.4	162
**Trux-OMeTAD** ^*b*^	23.2	1.02	0.79	18.6	16.3	149
**ICTH2**	24.78	1.03	0.735	18.75	14.74	159
**TAPC** ^*b*^	22.32	1.04	0.811	18.8	12.9	153
**FA-CN**	21.71	1.13	0.77	18.9	19.2	158
**KR321**	21.70	1.13	0.78	19.03	19.01	157
**YN2**	23.15	1.11	0.75	19.27	17.80	156

^*a*^Doped spiro-OMeTAD or PEDOT:PSS was used as the reference in most of the cases.

^*b*^p–i–n device architecture.

^*c*^Planar device architecture.

**Table 3:** Photovoltaic performance of the perovskite devices employed with best performing dopant-free polymeric HTMs sorted by PCE of the champion device

ID	*J*_sc_ (mA cm^–2^)	*V*_oc_ (V)	FF	PCE (%)	Ref. PCE^*a*^ (%)	Lit. ref.
**P3CT-Na** ^*b*^	21.3	1.05	0.75	18.3	—	165
**Asy-PBTBDT**	22.4	1.11	0.732	18.3	18.5	169
**PTB7** ^*c*^	22.6	1.13	0.71	18.5	13	168
**pDPP5T-2** ^*c*^	21.5	1.15	0.76	18.9	13	168
**PCDTBT1** ^*c*^	22.2	1.1	0.782	19.1	19.4	171
**P3CT-CH_3_NH_2_** ^*b*^	22.2	1.09	0.81	19.6	—	165
**DTB**	25.5	1.10	0.698	19.68	19.81	167
**TB(MA)** ^*b*^	23.45	1.08	0.78	19.76	16.93	166
**PDBT-T1** ^*c*^	22.2	1.17	0.77	19.8	13	168
**PTEG** ^*c*^	22.5	1.14	0.77	19.8	17.9	170
**PDCBT** ^*c*^	22.7	1.17	0.80	21.2	13	168

^*a*^Doped spiro-OMeTAD or PTAA was used as the reference in most of the cases.

^*b*^p–i–n device architecture.

^*c*^Planar device architecture.

So far, D–A type and branched structures have been proven to be two major design strategies to prepare high performance dopant-free HTMs. Such molecules enable better intermolecular interactions for the increase of the charge mobility. In general, the doping process is not required for efficient hole transport and collection if the HTM can exhibit a hole mobility as high as 10^–4^–10^–3^ cm^2^ V^–1^ s^–1^. Further optimization of these types of HTMs will undoubtedly lead to a PCE above 20% with excellent stability.

### Polymeric dopant-free HTMs

Polymers are potentially attractive HTM candidates in perovskite solar cells since they typically present good stability, processability, mechanical flexibility and film uniformity with tunable energy levels. Both high density and hydrophobicity of polymers are also very important for protecting the perovskite layer from moisture in ambient environments, therefore enhancing PSC lifetime, as discussed previously.

**Fig. 14:**
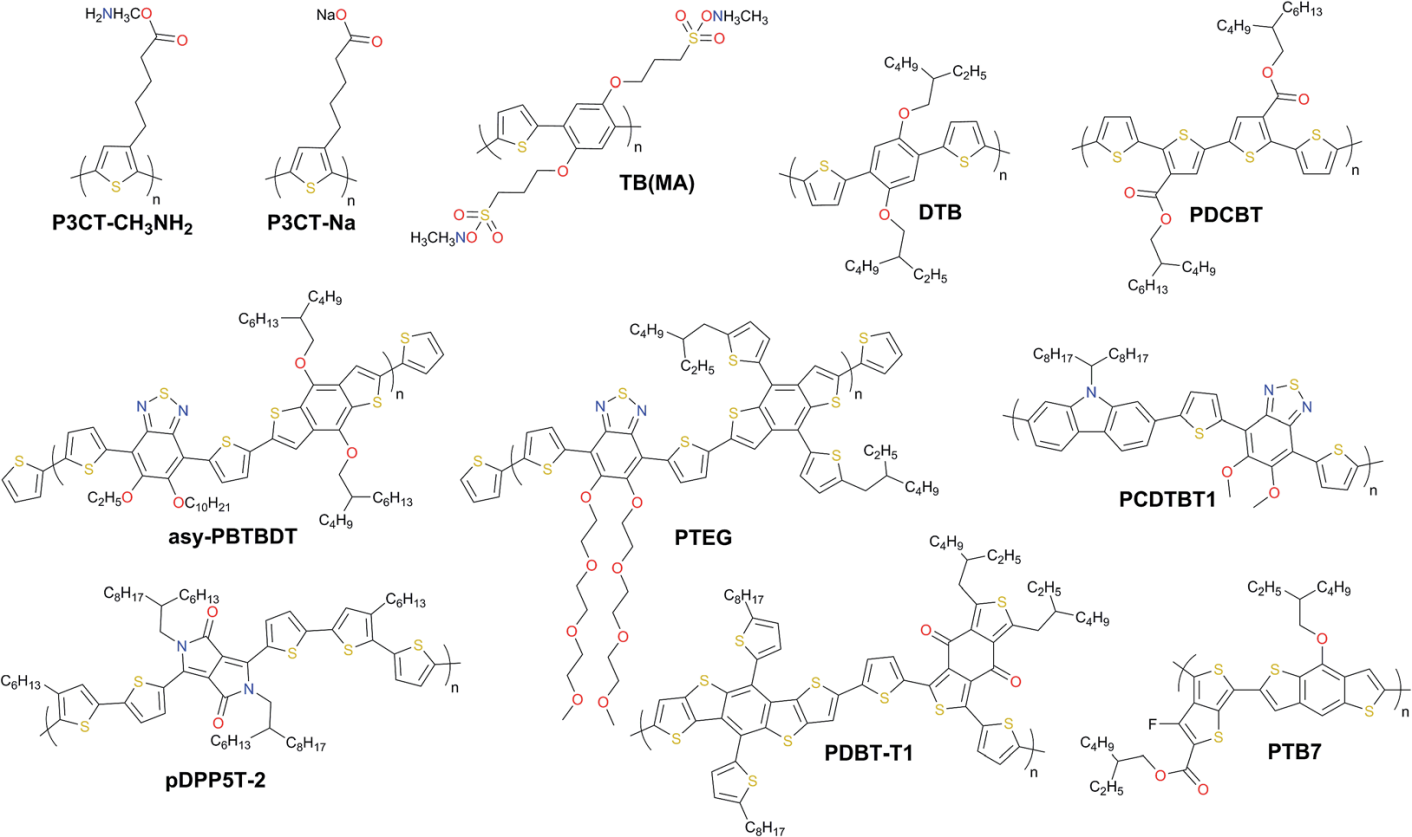
Chemical structures of the most efficient dopant-free polymeric hole transporting materials applied in PSCs.

Three different kinds of polymers are mostly used as HTMs for perovskite solar cells including homopolymer, copolymer, and D–A copolymer types. The class of polymeric HTMs has recently been found to be very effective for highly efficient and stable PSCs and is emerging very quickly. The chemical structures of the most efficient dopant-free polymeric hole transporting materials applied in PSCs are listed in Fig. 14 and Table 3.

The homopolymer-based polyelectrolytes **P3CT-CH_3_NH_2_** and **P3CT-Na** containing π-delocalized backbones with charged pendant groups were reported by Li *et al.*^165^ When using **P3CT-CH_3_NH_2_** to replace **P3CT-Na**, a much smoother film was obtained, which is important for the growth of a uniform perovskite layer on top in the inverted PSC configuration. In addition, the hole mobility is also improved in **P3CT-CH_3_NH_2_**, facilitating hole collection. By replacing **P3CT-Na** with **P3CT-CH_3_NH_2_**, the PCE has been greatly improved from 18.3 to 19.6%. Very recently, a similar strategy using side-chains end-capped by CH_3_NH_3_^+^ cations was reported by Zhang and co-workers.^166^ A conjugated polyelectrolyte termed **TB(MA)**, whose backbone is composed of 2,5-dialkoxy-1,4-phenylene and thiophene units showed a very high PCE of up to 19.76% in inverted PSCs.

**Fig. 15:**
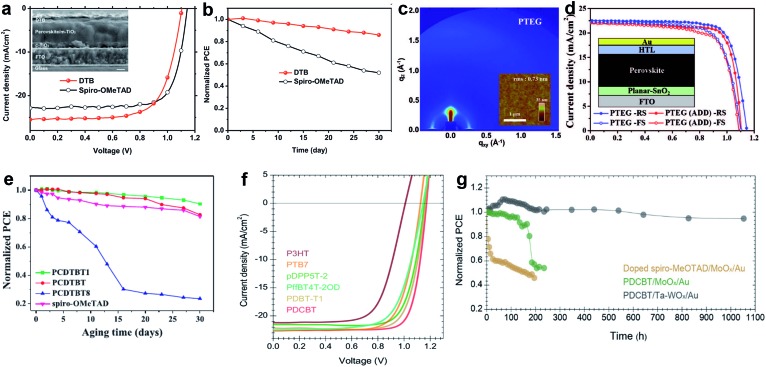
(a) *J*–*V* characteristics of **DTB** with a cross-sectional SEM image in the inset (scale bar: 200 nm); (b) evolution of PCE in air for PSC devices based on **DTB**. Reprinted with permission from ref. 167. (c) Grazing-incidence wide-angle X-ray scattering (GIWAXS) and atomic force microscopy (AFM) images of **PTEG**; (d) *J*–*V* curves for **PTEG** devices (planar-SnO_2_). Reprinted with permission from ref. 170. (e) The evolution of PCE for epoxy-encapsulated devices incorporating the **PCDTBT1** HTM that have been stored under ambient conditions for a duration of 30 days. Reprinted with permission from ref. 171. (f) The *J*–*V* characteristics of perovskite solar cells with different polymer HTMs under AM 1.5G illumination; (g) unencapsulated device photostability tests under continuous one sun illumination in a home-built chamber filled with N_2_. PCE variation of the HTM stacks based on a C_60_-SAM with strong binding between the phosphonic acid and the oxide surface as the ETM: spiro-MeOTAD/MoO_*x*_/Au (yellow), **PDCBT**/MoO_*x*_/Au (green) and **PDCBT**/Ta-WO_*x*_/Au (black). Reprinted with permission from ref. 168.

The edge-on alternating copolymer **DTB** with a rather simple structure composed of 2,5-dialkoxy-1,4-phenylene units and bithiophene units, where the alkoxyl side-chains are optimized endowing the polymer with both sufficient solubility in common solvents and an edge-on orientation atop perovskite was very recently reported by Zhang and co-workers. A high hole mobility of 1.0 × 10^–4^ cm^2^ V^–1^ s^–1^ and lamellar packing of polymer chains led to a stabilized PCE of 19.68% with an ultrahigh short-circuit current density of 25.50 mA cm^–2^ (Fig. 15a and b).^167^

Hou *et al.* reported a strategy to use tantalum doped tungsten oxide (Ta-WO_*x*_)/**PDCBT** conjugated polymer multilayer, which offer a surprisingly small interface barrier and form quasi-ohmic contacts leading to an impressive PCE of 21.2% combined with over 1000 hours of light stability (Fig. 15f and g).^168^

D–A type copolymers have been found to be very effective dopant-free HTMs as such a design strategy can ensure high hole mobility and enhanced device stability by rationally implementing different donors and acceptors. In addition, the introduction of suitable side chains into their conjugated backbones may form two-dimensional polymers with effectively tuned morphological properties.

Lee *et al.* reported a D–A type copolymer **asy-PBTBDT** comprising benzo[1,2-*b*:4,5:*b*′]dithiophene (BDT) and benzothiadiazole (BT) as donor and acceptor units, respectively.^169^ Introduction of asymmetric alkoxy substituents on BT units produced irregularity within a repeating unit of the polymer resulting not only in its solubility in common organic solvents, but also in its high solubility in the green solvent 2-methylanisole, which is a known food additive. The best device employing dopant-free **asy-PBTBDT** achieved a PCE of 18.3%, which is equivalent to the results obtained using the best control device employing doped spiro-OMeTAD. The performance was significantly improved using another BDT and BT based copolymer **PTEG** as reported by Kim and co-workers.^170^ Here, side chains were further engineered by introducing tetraethylene glycol (TEG) chains on BT units. TEG groups improve solubility by enhancing dipole–dipole interactions with the solvent. Moreover, flexible TEG groups can affect hole mobility by preventing backbone twisting and increasing π–π stacking. An improvement in the contact between perovskite and **PTEG** occurs because the surface energy of TEG groups is higher than that of alkyl groups. A dopant-free **PTEG**-based device achieved an impressive efficiency of 19.8% with a *J*_SC_ of 22.5 mA cm^–2^, a *V*_OC_ of 1.14 V, and a FF of 0.77 in planar PSCs (Fig. 15c and d). Cai *et al.* reported three HTMs comprising a carbazole donor moiety flanked by two thienyl groups that are co-polymerized with a pristine or functionalized BT unit with two methoxy or octyloxy substituents, resulting in **PCDTBT**, **PCDTBT1**, and **PCDTBT8**.^171^ It was found that the device performance strongly depends on the chemical structure of the conjugated polymer side chains. When **PCDTBT1** with two methoxy units on the BT unit was employed as the dopant-free HTM, the champion device efficiency was 19.1%, while the unsubstituted and octyloxy analogues gave PCEs of only 17.1% and 15.4%, respectively. Moreover, **PCDTBT1**-based devices show remarkable stability, retaining 90% of their original PCEs after storage for more than 30 days. This is attributed to their hydrophobic nature compared to that of spiro-OMeTAD as shown in Fig. 15e.

Various D–A type HTMs such as **PTB7**, **pDPP5T-2**, and **PDBT-T1** have also been screened for their device architecture with a Ta-WO_*x*_ doped interface delivering efficiencies of 18.5%, 18.9%, and 19.8%, respectively (Fig. 15f).^168^

Dopant-free polymers have recently been shown to achieve both high efficiency and device stability; however, they typically suffer from a few drawbacks such as batch-to-batch reproducibility, purity, and high synthetic cost. Another hot approach lies in crosslinking of small molecule HTMs after/before deposition of the perovskite layer. In this sense, the crosslinked-HTM exhibits higher hole mobility and morphological stability, as well as enhancing hydrophobicity, protecting the perovskite layer from decomposition. The major challenge here is the exploration of suitable crosslinking chemistry and functional groups.

## Conclusions and outlook

This article overviews the highest performance doped and dopant-free HTMs, selected from the HTM database, which includes over 400 publications on the inorganic and organic molecular and polymeric hole transporting materials published until 2019. These hand-picked flagship molecules demonstrate how small changes in the molecular structure such as different atoms and different functional groups, and changes in substitution positions or the length of the π-conjugated systems can result in significant changes in the properties of the hole transporting materials and performance of perovskite solar cell devices. We divided our HTM library into three categories: molecular doped HTMs, molecular dopant-free HTMs and polymeric dopant-free HTMs. The photovoltaic performances of the champion perovskite devices of selected molecules were compared with the most common reference materials such as spiro-OMeTAD, PTAA, and PEDOT:PSS.

We note that for efficient device operation, the well-aligned energy level of HTMs with that of perovskite is crucial: the HOMO level must be less negative than that of perovskite for efficient hole injection, while the high-lying LUMO level will ensure low electron affinity enabling beneficial electron blocking properties. An ideal HTM candidate should also have high hole mobility to reduce losses during hole transport to the contact, as well as sufficient conductivity. The HTM has to be well soluble and should not require strong polar and protic solvents, as such solvents may dissolve the perovskite layer. Furthermore, since perovskites are known to be sensitive to moisture and oxygen, the hole transporting layer should act as protection against air and prevent the diffusion of external moieties; therefore high thermal and photochemical stability, as well as high hydrophobicity, are desirable properties of HTMs to resist degradation and ensure long-term operation. On the other hand, to ensure low-cost perovskite solar cell manufacturing, an ideal HTM candidate has to be easily affordable by simple synthetic schemes with a minimized number of steps and easy workup and purification procedures for cost-effective upscaling.

The quest to design the best hole transporting molecule with well-aligned energy levels, sufficient solubility, and high hole mobility and conductivity opens the door to better-performing perovskite solar cell devices. On the other hand, while perovskite solar cells are making great strides toward commercialization, the use of highly stable hole transport molecules is crucial for the long-term stability of the overall device.

Tremendous effort has been made to develop new alternative low-cost and efficient HTMs to replace spiro-OMeTAD, and organic small-molecule HTMs are the most widely developed group in PSCs owing to their synthetic variety, tunable properties, high purity, and simple solution processing. However, most of the reported high-efficiency doped HTMs suffer from rapid decomposition and are limited by a very short operation lifetime of up to a few months due to the dopant combination of the cobalt complex (FK209) and lithium bis(trifluoromethanesulfonyl)imide (LiTFSI) with the additive of 4-*tert*-butylpyridine (*t*BP) as the morphology controller.

In this context, a unique solution to eliminate the negative influence of chemical dopants on the stability of the entire perovskite solar device is to utilize dopant-free HTMs. Besides the outstanding contribution to the device stability by dopant-free HTMs, these materials have become very attractive because of their improvement in terms of power conversion efficiency of perovskite devices. Two main design strategies of dopant-free HTMs have been introduced so far to achieve the highest performance. D–A type and branched structures provide better intermolecular interactions towards increased charge mobility.

A novel approach using a hole-transporting self-assembled monolayer (SAM) as the dopant-free hole-selective contact in highly efficient p–i–n PSCs has been demonstrated due to its anchoring group binding to the ITO surface. Further gains in efficiency and stability are expected with SAM optimization using molecular and compositional engineering.

Another important approach is dopant-free polymers, which has recently been shown to provide both high PCE and device stability, although there are several drawbacks: reproducibility, low purity, and high synthetic cost. Lastly, the cross-linking of small-molecule HTMs before/after deposition of the perovskite layer is a promising direction in this field. The advantage of this approach is that the crosslinked-HTM exhibits higher hole mobility and morphological stability, as well as increased hydrophobicity that may protect the perovskite layer from decomposition. However, a deeper investigation towards appropriate crosslinking chemistry and functional groups is required.

Although several reviews on HTMs have been published, progress on dopant-free HTMs needs to be reviewed and analyzed facilitating further development of molecular and polymeric dopant-free HTMs and alternative chemical approaches such as ‘‘click chemistry’’ inspired crosslinking and self-assembled monolayer formation for the inevitable replacement of doped spiro-OMeTAD. We hope that this mini-review cataloging the library of the most efficient molecules will be of use to the community and help to develop solutions for more efficient and innovative compounds.

## Conflicts of interest

There are no conflicts to declare.
